# Big Data Analysis of Genes Associated With Neuropsychiatric Disorders in an Alzheimer’s Disease Animal Model

**DOI:** 10.3389/fnins.2018.00407

**Published:** 2018-06-15

**Authors:** Suji Ham, Tae K. Kim, Heeok Hong, Yong S. Kim, Ya-Ping Tang, Heh-In Im

**Affiliations:** ^1^Convergence Research Center for Diagnosis, Treatment and Care System of Dementia, Korea Institute of Science and Technology (KIST), Seoul, South Korea; ^2^Division of Bio-Medical Science & Technology, KIST School, University of Science and Technology, Seoul, South Korea; ^3^Department of Biology, Boston University, Boston, MA, United States; ^4^Department of Medical Science, Graduate School of Medicine, Konkuk University, Seoul, South Korea; ^5^Department of Pharmacology, Seoul National University College of Medicine, Seoul National University, Seoul, South Korea; ^6^Neuroscience Center of Excellence, Louisiana State University Health Sciences Center New Orleans, New Orleans, LA, United States; ^7^Center for Neuroscience, Brain Science Institute, Korea Institute of Science and Technology (KIST), Seoul, South Korea

**Keywords:** Alzheimer’s disease, big data, microarray, neuropsychiatric symptoms, bipolar disorder, schizophrenia, major depression disorder

## Abstract

Alzheimer’s disease is a neurodegenerative disease characterized by the impairment of cognitive function and loss of memory, affecting millions of individuals worldwide. With the dramatic increase in the prevalence of Alzheimer’s disease, it is expected to impose extensive public health and economic burden. However, this burden is particularly heavy on the caregivers of Alzheimer’s disease patients eliciting neuropsychiatric symptoms that include mood swings, hallucinations, and depression. Interestingly, these neuropsychiatric symptoms are shared across symptoms of bipolar disorder, schizophrenia, and major depression disorder. Despite the similarities in symptomatology, comorbidities of Alzheimer’s disease and these neuropsychiatric disorders have not been studied in the Alzheimer’s disease model. Here, we explore the comprehensive changes in gene expression of genes that are associated with bipolar disorder, schizophrenia, and major depression disorder through the microarray of an Alzheimer’s disease animal model, the forebrain specific PSEN double knockout mouse. To analyze the genes related with these three neuropsychiatric disorders within the scope of our microarray data, we used selected 1207 of a total of 45,037 genes that satisfied our selection criteria. These genes were selected on the basis of 14 Gene Ontology terms significantly relevant with the three disorders which were identified by previous research conducted by the Psychiatric Genomics Consortium. Our study revealed that the forebrain specific deletion of Alzheimer’s disease genes can significantly alter neuropsychiatric disorder associated genes. Most importantly, most of these significantly altered genes were found to be involved with schizophrenia. Taken together, we suggest that the synaptic dysfunction by mutation of Alzheimer’s disease genes can lead to the manifestation of not only memory loss and impairments in cognition, but also neuropsychiatric symptoms.

## Introduction

Psychiatric disorders such as bipolar disorder (BIP), schizophrenia (SCZ), and major depressive disorder (MDD) are classified based on neuropsychiatric symptoms (NPS), and these diseases remain to be the most prevalent among others. Patients suffering from NPS have been documented to experience symptoms that are representative of these disorders such as mood swings, hallucinations, and depression. The prevalence of BIP, SCZ, and MDD is considerable worldwide and also in the United States, with its 12-month prevalence amounting to 2.6, 1.1, and 6.7%, respectively (Regier et al., 1993; Kessler et al., 2005). Although the etiology of NPS is not yet clear, it is accepted that NPS are caused by alterations within the brain, namely changes in gene expression.

NPS refer to non-cognitive symptoms of apathy, anxiety, agitation, aggression, depression, hallucinations, and delusions, unlike cognitive symptoms of Alzheimer’s disease (AD) such as loss of memory and reduced executive functions. NPS represent a high proportion of the global burden of disease, primarily due to the lack of effective preventive and therapeutic methods for treating NPS (WHO, 2001). Interestingly, AD is significantly similar in NPS with BIP, SCZ, and MDD. AD patients have reported frequent experience of NPS, which has been found to have 80–90% prevalence across patients (Steinberg et al., 2004; Nowrangi et al., 2015). AD patients with NPS also show an increase in the rate of hospitalization and mortality while its presence cuts life expectancy by 10–20 years (Sweet et al., 2003; WHO, 2016).

Similarly, AD animals have also been found to elicit NPS in a number of transgenic models. Well-characterized AD transgenic models such as the hAPP/PS1 mouse has been observed to exhibit aggressive behavior, while TgCRND8 mice carrying Swedish and Indiana APP mutations were reported to demonstrate increased auditory response and reduced prepulse inhibition responses (McCool et al., 2003; Pugh et al., 2007). Notably, previous research has indicated that mice with forebrain specific presenilin 1 and 2 knockout (PSEN dKO) may possess a variety of NPS ranging from depression, apathy, and aggressive behavior – which mirrors many of the NPS present in AD patients (Yan et al., 2013).

In order to discover the molecular mechanisms underlying AD and psychiatric disorders, high throughput techniques providing comprehensive results of the transcriptome – such as microarrays – are utilized to identify genes responsible for pathogenesis (Mirnics et al., 2000; Hakak et al., 2001; Kang et al., 2007). Currently, most microarray studies screen for genes that significantly change compared to controls, and analyze the interaction of individual genes in pathways and networks to indirectly reveal the phenotype of these genes (Li and Chen, 2014). Similarly, previous microarray studies using AD models have selected genes and subsequently performed pathway and network analysis to explain the manifestation of cognitive deficits in AD. However, in our study, we sought to identify significantly altered genes within the gene ontology (GO) gene pools associated with psychiatric disorders (BIP, SCZ, and MDD), and to explain whether NPS in AD may have resulted from the changes in these genes.

To obtain the GO gene pools of psychiatric disorders (BIP, SCZ, and MDD), we cited a genomic-wide association study (GWAS) that was based on single nucleotide polymorphisms (SNP) (Network and Pathway Analysis Subgroup of Psychiatric Genomics Consortium, 2015). The study suggested 20 pathways relevant to psychiatric disorders such as BIP, SCZ, and MDD. As we believed that GO terms from SNP studies could represent the symptoms of BIP, SCZ, and MDD, we used 14 GO terms that were enriched with BIP, SCZ, and MDD susceptibility alleles. We observed significant expression changes of genes in the cortex and hippocampus of PSEN dKO mice, brain regions where neurodegeneration occurs in AD. These genetic alterations were not only related to cognitive decline in AD, but also associated with NPS observed in AD.

## Results

### The BIP, SCZ, and MDD Related Genes Were Significantly Changed in AD Animal Model

**FIGURE 1:**
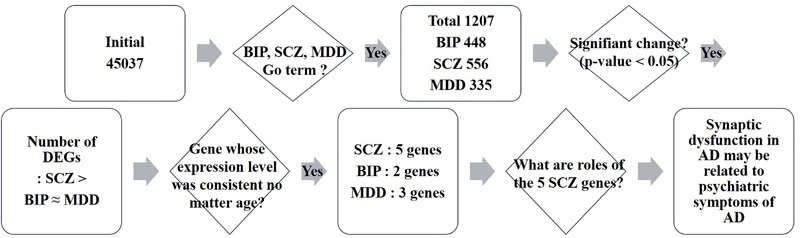
Schematic flow of big data analysis. Rectangles indicate the result of each step of microarray analysis. The phrase “Initial 45037” denotes the number of probes in the microarray. Rhombus denotes a criteria or question asked in each step. DEG: differentially expressed genes.

We investigated whether BIP, SCZ, and MDD associated genes were changed in an AD mouse model, the PSEN dKO mouse, through microarray analysis. For this, we implemented two processes. First, we filtered BIP, SCZ, and MDD associated genes from all probes included within the AD microarray data. We used genes within the 13 GO terms shown in **Table 1**. These GO terms had already been reported to show a close relationship with SNP of BIP, SCZ, and MDD patients in the study conducted by the Network and Pathway Analysis Subgroup of the Psychiatric Genomics Consortium (PGC) (Network and Pathway Analysis Subgroup of Psychiatric Genomics Consortium, 2015). As a result, the number of mRNA probes that we wanted to focus on decreased from 45,037 to 2558. The 2558 mRNA probes were specific to 1207 genes due to overlapping probes for a particular gene (**Figure 1**). Second, we selected statistically significant altered genes at two age points, and in the cortex and hippocampus of AD mice. We chose two time points (7 and 18 months) at which several characteristics of AD such as increased amyloid beta peptide, hyper-phosphorylated tau, neuronal loss, and memory deficit were known to be clearly observed (Ham et al., 2017). The two brain regions had been reported to be involved in both memory loss of AD and NPS (Godsil et al., 2013). Among these 1207 genes that were relevant to BIP, SCZ, and MDD, 93, 111, and 69 genes associated with BIP, SCZ, and MDD, were significantly altered in AD mice (**Supplementary Material S1**). The number of differentially expressed genes (DEG) in PSEN dKO and wild-type (WT) mice was 2261 (data not shown), indicating that the BIP, SCZ, and MDD related genes accounted for 4.1, 4.9, and 3% of the total number DEGs, respectively. We also performed the kyoto encyclopedia of genes and genomes (KEGG) pathway analysis with the previously identified 93, 111, and 69 genes of BIP, SCZ, and MDD (**Supplementary Material S2**).

**Table 1:** GO terms of bipolar disorder, schizophrenia, and major depression disorder.

BIP		SCZ		MDD	
GO term	The number of genes within each GO term	GO term	The number of genes within each GO term	GO term	The number of genes within each GO term
Histone H3-K4 methylation	32	Postsynaptic density	205	Protein phosphatase type 2A regulator activity	8
(Chromosomal) synapsis	23	Postsynaptic membrane	207	Cell junction organization	137
Regulation of anatomical structure size	54	Dendritic spine	116	Apical junction assembly	122
Chromosome organization involved in meiosis	362	Histone H3-K4 methylation	32	Cell-cell junction organization	67
–	–	Axon part	192	Regulation of histone modification	135
The total number of genes within BIP GO terms	448 (23)	The total number of genes within SCZ GO terms	556 (196)	The total number of genes within MDD GO terms	335 (134)

*GO terms that have known to be associated with bipolar disorder, schizophrenia, and major depression disorder. The number of genes associated within each GO term and the total number of genes in the disorder associated GO terms is represented along with overlapping genes across each GO term. The number of overlapping gene across each GO term associated with a disorder (BIP, SCZ, and MDD) is indicated in parentheses and the total number of genes in all GO terms is also specified. Connection of these GO terms with the three neuropsychiatric disorders have been reported by the Network and Pathway Analysis Subgroup of the PGC. BIP, bipolar disorder; SCZ, schizophrenia; MDD, major depression disorder.*

### The SCZ-Related Genes Than the BIP- and MDD-Related Genes Were Altered in AD Animal Model

**FIGURE 2:**
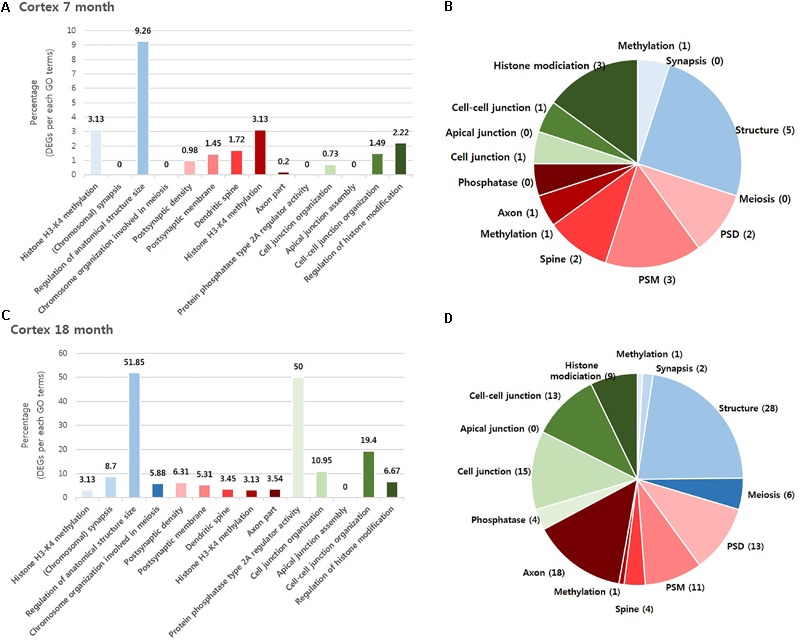
Overall comparison of significantly altered genes in the cortex of AD model within GO terms of BIP, SCZ, and MDD. Various shades of blue indicate BIP GO terms. Various shades of red indicate SCZ GO terms. Various shades of green indicate BIP terms. The bar graph represents the percent of significantly changed genes based on the total number of genes within each GO terms. The pie chart represents the number of significantly altered genes within each GO terms. **(A)** and **(B)** have resulted from analysis in the cortex of 7 month aged AD mice. **(C)** and **(D)** resulted from the cortex of 18-month old AD mice. Methylation: Histone H3-K4 methylation; Synapsis: (Chromosomal) synapsis; Structure: Regulation of anatomical structure size; Meiosis: Chromosome organization involved in meiosis; PSD: Postsynaptic density; PSM: Postsynaptic membrane; Spine: Dendritic spine; Axon: Axon part; Phosphatase: Protein phosphatase type 2A regulator activity; Cell junction : Cell junction organization; Apical junction: Apical junction assembly; Cell-cell junction: Cell-cell junction organization; Histone modification: Regulation of histone modification.

**FIGURE 3:**
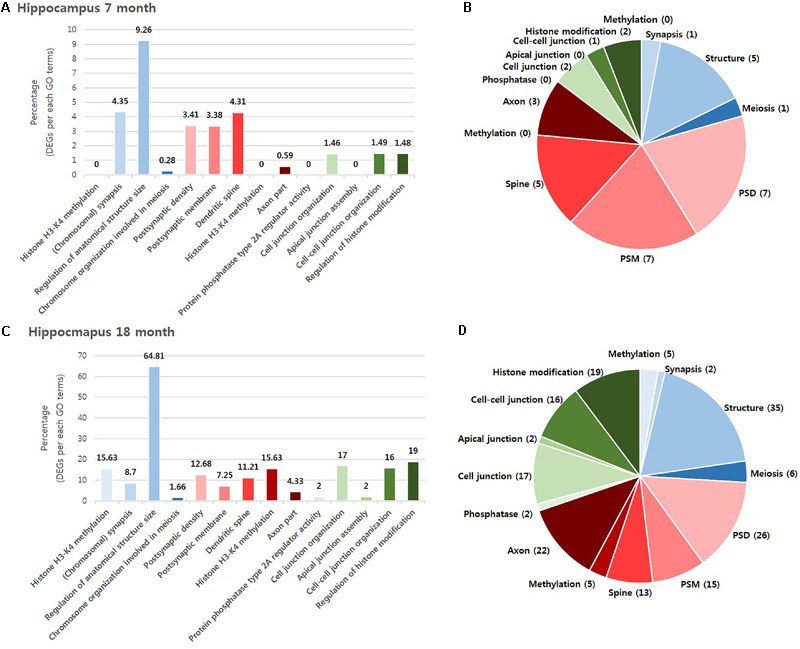
Overall comparison of significantly altered genes in the hippocampus of AD model within GO terms of BIP, SCZ, and MDD. Various shades of blue indicate BIP GO terms. Various shades of red indicates SCZ GO terms. Various shades of green indicate BIP terms. The bar graph represents the percent of significantly changed genes based on the total number of genes within each GO terms. The pie chart represent the number of significantly altered genes within each GO terms. **(A)** and **(B)** resulted from analysis in the hippocampus of 7-month old AD mice. **(C)** and **(D)** resulted from the hippocampus of 18-month old AD mice. Methylation: Histone H3-K4 methylation; Synapsis: (Chromosomal) synapsis; Structure: Regulation of anatomical structure size; Meiosis: Chromosome organization involved in meiosis; PSD: Postsynaptic density; PSM: Postsynaptic membrane; Spine: Dendritic spine; Axon: Axon part; Phosphatase: Protein phosphatase type 2A regulator activity; Cell junction : Cell junction organization; Apical junction: Apical junction assembly; Cell-cell junction: Cell-cell junction organization; Histone modification: Regulation of histone modification.

To investigate which NPS was most involved with AD, we visualized the “percentage” and “number” of significantly altered genes within the BIP, SCZ, and MDD GO terms by bar graph and pie chart (**Figures 2, 3**). In the cortex of 7-month old AD mice, SCZ associated genes did not account for a largest proportion of the altered genes (**Figure 2A**). However, the number of genes associated to SCZ was higher than that of BIP and MDD related genes (**Figure 2B**). The proportion of SCZ associated genes was decreased in the cortex of 18-month old AD mice (**Figure 2C**), but the number of genes related to SCZ was still higher than that of BIP and MDD related genes (**Figure 2D**). The changes of these genes in hippocampus were similar to that in the cortex. Although SCZ related genes did not account for a large portion in the hippocampus of 7 and 18- month old AD mice (**Figures 3A,B**), the number of genes related to SCZ was also higher than that of BIP and MDD related genes in hippocampus of both 7- and 18-month old AD mice (**Figures 3C,D**).

We aimed to select the most influential psychiatric symptom in AD via GO terms of BIP, SCZ, and MDD. The selection process required a condition to be satisfied: the three neuropsychiatric disorders should have distinct GO terms. Among the 13 GO terms that we used, the GO terms for epigenetics (Histone H3-K4 methylation in BIP and SCZ, Regulation of histone modification in MDD) were shared between the three disorders (**Table 1**). On the other hand, the differences between the GO terms of the three diseases were found to be clear. The GO terms of BIP were related to chromosome, those of SCZ were related to the synapse, and those of MDD were related to the cell junction (**Table 1**). Therefore, due to these differences between GO terms of three GO terms this conferred suitability for selecting one neuropsychiatric disorder.

We aimed to determine the neuropsychiatric disorder that was most relevant with AD among BIP, SCZ, and MDD. There were two possible criteria for this decision. One was “percentage,” which was defined as the ratio of DEG to the total number genes of each GO term, and the other was the “number” of DEGs within a neuropsychiatric disorder-specific GO term. We selected the “number” of DEGs as, for each of the GO terms that were ranked in accordance to the “percentage” of gene expression changed, the “percentage” imposed GO term-specific meaning which in turn may introduce confounding variables. However, this GO term-specific meaning was not inferred when ranking the disorder by the “number” of DEG, and this also allowed an objective, comprehensive determination of the most relevant neuropsychiatric disorder associated with AD. As a result, our data indicates that SCZ was the most relevant neuropsychiatric disorder associated with AD (**Table 2**).

**Table 2:** Ranking by percentages and numbers of significantly changed genes within GO terms of BIP, SCZ, and MDD.

	GO terms	Cortex				Hippocampus			
		7 months		18 months		7 months		18 months	
		Number	Percentage	Number	Percentage	Number	Percentage	Number	Percentage
BIP	Histone H3-K4 methylation	1	3.13 (1/32)	1	3.13 (1/32)	0	0.00 (0/32)	5	15.63 (5/32)
	(Chromosomal) synapsis	0	0.00 (0/23)	2	8.70 (2/23)	1	4.35 (1/23)	2	8.70 (2/23)
	Regulation of anatomical structure size	5	9.26 (5/54)	28	51.85 (28/54)	5	9.26 (5/54)	35	64.81 (35/54)
	Chromosome organization involved in meiosis	0	0.00 (0/362)	6	1.66 (6/362)	1	0.28 (1/362)	6	1.66 (6/362)
	**Total**	**6**	**1.34 (6/448)**	**40**	**8.93 (40/448)**	**6**	**1.34 (6/448)**	**57**	**12.72 (57/448)**
SCZ	Postsynaptic density	2	0.98 (2/205)	13	6.34 (13/205)	7	3.41 (7/205)	26	12.68 (26/205)
	Postsynaptic membrane	3	1.45 (3/207)	11	5.31 (11/207)	7	3.38 (7/207)	15	7.25 (15/207)
	Dendritic spine	2	1.72 (2/116)	4	3.45 (4/116)	5	4.31 (5/116)	13	11.21 (13/116)
	Histone H3-K4 methylation	1	3.13 (1/32)	1	3.13 (1/32)	0	0.00 (0/32)	5	15.63 (5/32)
	Axon part	1	0.20 (1/192)	18	3.54 (18/192)	3	0.59 (3/192)	22	4.33 (22/192)
	**Total**	**^∗^8**	**^∗^1.44 (8/556)**	**^∗^53**	**9.53 (53/556)**	**^∗^14**	**^∗^2.52**	**80**	**^∗^14.39 (80/556)**
MDD	Protein phosphatase type 2A regulator activity	0	0.00 (0/8)	4	50.00 (4/8)	0	0.00 (0/8)	2	25.00 (2/8)
	Cell junction organization	1	0.73 (1/137)	15	10.95 (15/137)	2	1.46 (2/137)	17	12.41 (17/137)
	Apical junction assembly	0	0.00 (0/122)	0	0.00 (0/122)	0	0.00 (0/122)	2	1.64 (2/122)
	Cell-cell junction organization	1	1.49 (1/67)	13	19.40 (13/67)	1	1.49 (1/67)	16	23.88 (16/67)
	Regulation of histone modification	3	2.22 (3/135)	9	6.67 (9/135)	2	1.48 (2/135)	19	14.07 (19/135)
	**Total**	**4**	**1.19 (4/335)**	**39**	**^∗^11.64 (39/335)**	**4**	**1.19 (4/335)**	**48**	**14.33 (48/335)**

*The three psychiatric disorders, BIP, SCZ, and MDD, are ranked by the percentage and number of genes. In each of these disorders, GO terms are specified, and the number of significantly changed genes and the percentage is indicated for each region and time point. Percent indicates the ratio of the number of significantly changed genes to the number of genes within each GO Term. The total refers to the total number of genes included in each of the GO terms for each of the three disorders. Star denotes the highest ranking disorder determined by the highest total number of significantly altered genes or percentage at each region and time point.*

### Prominently Changed SCZ-Related Genes Were Mainly Involved in Pre- and Post-synaptic Functions

**FIGURE 4:**
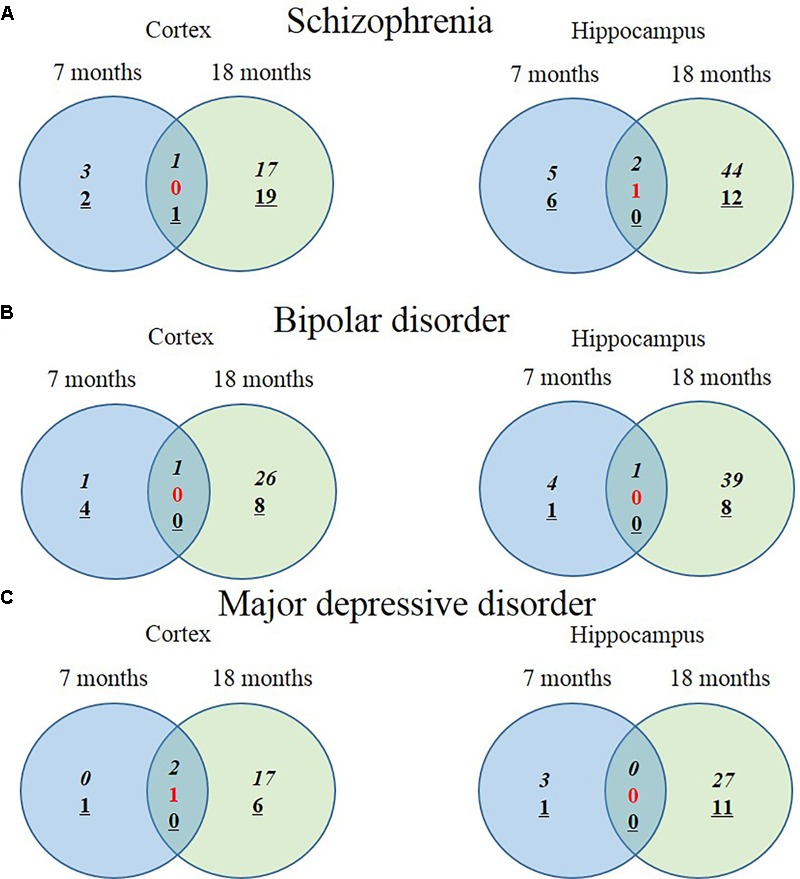
Venn diagram displaying numbers of upregulated and downregulated SCZ-associated genes in AD animal model. The left Venn diagrams show differently expressed cortical mRNAs of AD mice compared to control mice and the right Venn diagrams show differently expressed hippocampal mRNAs of AD mice compared to control mice (*P*-value < 0.05, Fold change > 1.3). **(A)** The number of significantly altered genes within five GO terms associated with SCZ. **(B)** The number of significantly altered genes within four GO terms associated with BIP. **(C)** The number of significantly altered genes within five GO terms associated with MDD. Italicization denotes the number of up-regulated genes and an underline denotes the number of down-regulated genes. Numbers in red indicate the number of contra-regulated genes that are upregulated in one group and downregulated in another group.

**FIGURE 5:**
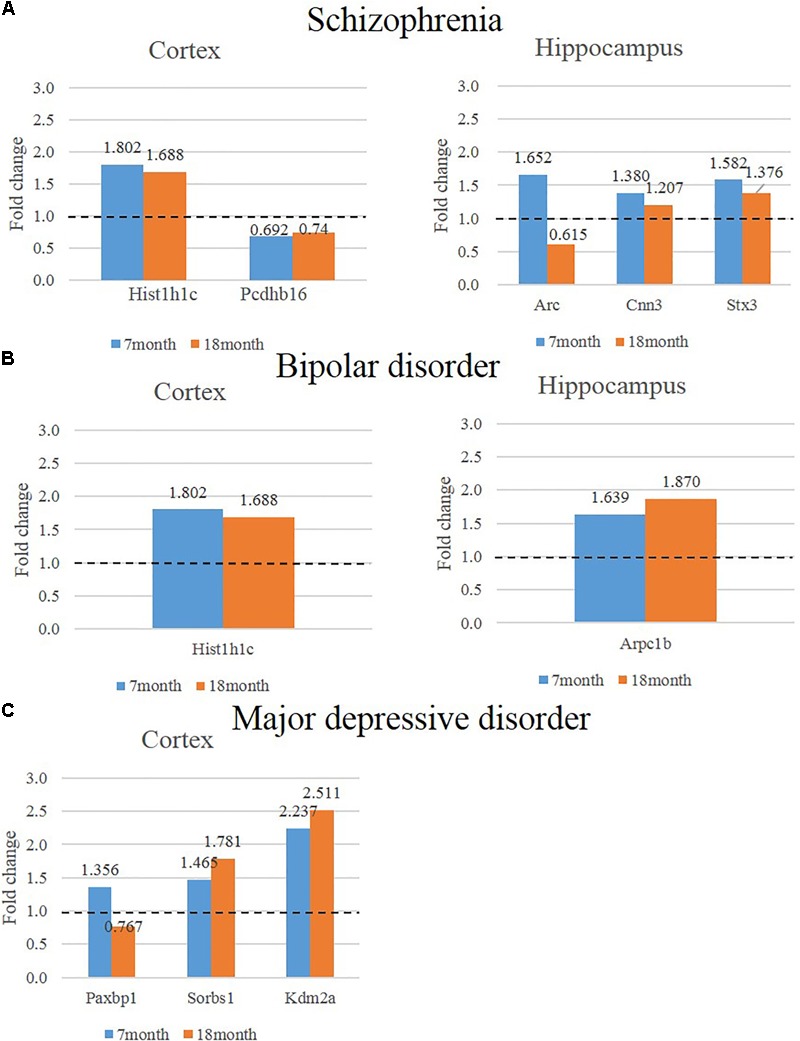
Expression of genes that show consistently significant alterations in two different ages (7, 18 months) of AD animal model. The left graph shows consistently altered cortical mRNAs in two different ages (7, 18 months) of AD mice compared to control mice and the graph on the right shows consistently altered cortical mRNAs in two different ages of AD mice compared to control (*P*-value < 0.05, Fold change > 1.3). **(A)** The expression of SCZ associated genes in the cortex is indicated in the bar graph on the left and hippocampus is indicated in the graph on the right. **(B)** The expression of BIP associated genes in the cortex is indicated in the bar graph on the left and hippocampus is indicated in the graph on the right. **(C)** The expression of MDD associated genes in the cortex is indicated in the bar graph on the left and hippocampus is indicated in the graph on the right.

**FIGURE 6:**
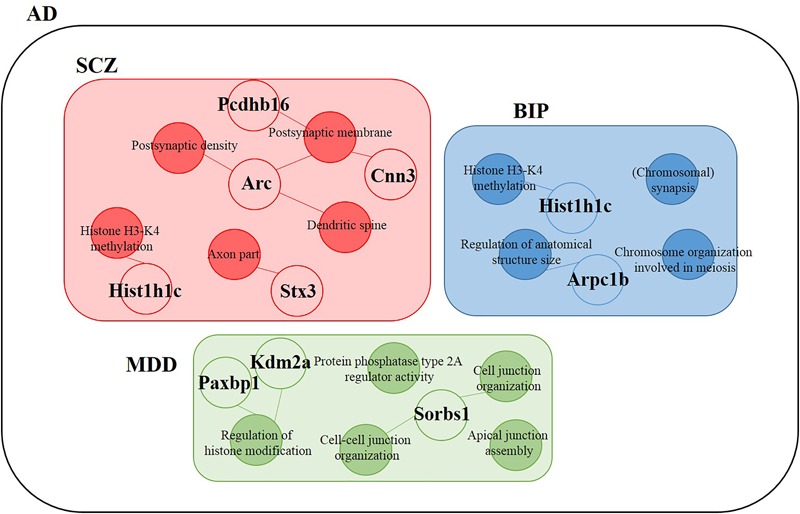
Differentially expressed genes within GO terms of BIP, SCZ, and MDD. The SCZ associated genes (*Hist1h1c, Pcdhb16, Arc, Cnn3*, and *Stx3*) belonged to Postsynaptic density (*Arc, Cnn3*), Postsynaptic membrane (*Pcdhb16, Arc*), Dendritic spine (*Arc, Cnn3*), Histone H3-K4 methylation (*Hist1h1c*), and Axon part (*Stx3*). The BIP associated genes (*Hist1h1c* and *Arpc1b*) belonged to Histone H3-K4 methylation and Regulation of anatomical structure size, respectively. The MDD-associated genes (*Paxbp1, Sorbs1*, and *Kdm2a*) belonged to Cell junction organization, Cell-cell junction organization, and Regulation of histone modification. Empty circles indicate genes and filled circles indicate GO terms. The lines between empty and filled circles denote that the gene belongs to the GO terms.

We hypothesized that 93 (BIP), 111 (SCZ), and 69 (MDD) genes with significant expression changes will show changed expression at both 7 and 18 months if they are truly associated to BIP, SCZ, or MDD. To identify genes that changed at both 7 and 18 months, we sought to discover sets of overlapping genes according to their regions and neuropsychiatric disorders (**Figure 4**). We denoted overlapping genes as “common genes” to differentiate from the aforementioned significantly altered genes. Common genes in SCZ were *Hist1h1c, Pcdhb16* in the cortex, and were *Arc, Cnn3*, and *Stx3* in the hippocampus (**Figure 5A**). Common genes in BIP were *Hist1h1c, Arpc1b* in the cortex and hippocampus, respectively (**Figure 5B**). In MDD, common genes were *Paxbp1, Sorbs1*, and *Kdm2a* in the cortex (**Figure 5C**). However, common genes were not found in the hippocampus for MDD. It is important to note that these common genes were specifically altered within the cortex or hippocampus (**Table 3**). To assess role of these genes, GO terms of common genes were represented in **Figure 6**. The *Pcdhb16, Arc*, and *Cnn3* among five SCZ genes were constituents within the GO terms of Postsynaptic density, Postsynaptic membrane, and Dendritic spine. These GO terms were primarily associated with pre- and post-synaptic functions. Consequently, SCZ-related genes in AD mice may be involved in synaptic dysfunction.

**Table 3:** Region specific expression of genes that showed consistently significant alterations in two different ages.

		Fold change			
		Cortex		Hippocampus	
		7 months	18 months	7 months	18 months
SCZ	Hist1h1c	1.802^∗^	1.688^∗^	1.536	1.175
	Pcdhb16	0.692^∗^	0.740^∗^	1.034	1.135
	Arc	1.512	0.422	1.652^∗^	0.615^∗^
	Cnn3	1.255	1.877	1.380^∗^	1.207^∗^
	Stx3	1.062	0.869	1.582^∗^	1.376^∗^
BIP	Hist1h1c	1.802^∗^	1.688^∗^	1.536	1.175
	Arpc1b	1.887	1.661	1.639^∗^	1.870^∗^
MDD	Paxbp1	1.356^∗^	0.767^∗^	1.337	1.020
	Sorbs1	1.465^∗^	1.781^∗^	1.161	1.368
	Kdm2a	2.237^∗^	2.511^∗^	2.229	1.755

*This table represents gene expressions that were specifically altered within the cortex or the hippocampus. Star denotes statistically significant changes in gene expression.*

## Discussion

This is the first study to attempt the analysis of gene expressions for NPS in a mouse model of AD. In the present study, we profiled mRNA expressions in the cortex and the hippocampus in comparison to the wild type group using GO terms related with BIP, SCZ, and MDD. By exploring the patterns of gene expressions, we found significant differences in BIP, SCZ, and MDD-related genes in AD model. In addition, we found that the genes in the hippocampus showed more dramatic changes than in the cortex. Furthermore, we discovered an interesting point. The SCZ-related genes were the most noticeable genes among the genes of the three disorders and the altered SCZ-related genes were mainly involved in synaptic function. Accordingly, this study may help to clarify underlying mechanisms of NPS in AD.

Here we found altered expression of neuropsychiatric disorder associated genes in AD model. As we believed that GO terms by SNP studies could represent the symptoms of BIP, SCZ, and MDD, we interpreted the changed expressions of these genes in AD as probable causes of BIP-like, SCZ-like, and MDD-like NPS in AD such as mood swings, hallucinations, and depression. Also, it is notable that the genes in the AD animal model are being investigated by using the results of human studies. Therefore, transcriptome analysis of this AD mouse model based on human studies allows for the application of results to NPS in AD patients in the future.

The cortex and hippocampus are brain regions show neurodegeneration in both AD patients and AD animal models (Saura et al., 2004). The AD animal model, PSEN dKO mice, showed earlier progression of neurodegeneration in the cortex than in the hippocampus, and gene expression changes at later stages were mainly observed in the hippocampus than in the cortex (Wines-Samuelson et al., 2010; Watanabe et al., 2014; Ham et al., 2017). Consistent with these previous studies, in this study, marked alterations in expressions of BIP, SCZ, and MDD related genes in the hippocampus were observed compared to the cortex due to old age of AD mice (7 and 18 month aged mice). An important point to consider is that it is easy to conceive that these brain regions are suitable for screening genes associated with memory loss and cognitive symptoms than NPS in AD. However, the cortex and hippocampus are brain regions associated with cognitive dysfunction and hallucination of BIP, SCZ, and MDD as well as memory and cognition functions of AD (Frodl et al., 2006; Amad et al., 2014). Therefore, the present investigation performed within the regions of cortex and hippocampus was adequate for NPS in AD. However, in order to confer higher accuracy in terms of regional association for NPS in AD, further studies will be needed within the regions of caudate putamen, nucleus accumbens, amygdala, and hypothalamus, as well as the cortex and hippocampus (Sun et al., 2013).

Schizophrenic symptoms such as hallucination and delusion occur considerably in AD to such an extent that new diagnosis, “AD with psychosis” (AD+P) has been formed (Murray et al., 2014). Those with AD+P account for 30–50% among all AD patients (Murray et al., 2014). On the other hand, depression is more common in AD than schizophrenic symptoms (about 50%) (Garcez et al., 2015). However, our results showed the lowest relationship between AD and depression. Interestingly, the most dominant changes in gene expression were observed in schizophrenia-related genes within both brain regions. It is important to note that schizophrenic symptoms are manifest usually in the later stages of AD whereas depressive symptoms appear especially during the early and middle stages of AD^1^^,^^2^. Consequently, due to using old aged AD mice in this study, SCZ-related genes than MDD-related genes in the AD model were differentially expressed compared to normal mice.

The SCZ associated genes were *Hist1h1c, Pcdhb16, Arc, Cnn3*, and *Stx3*. Among these genes, the protocadherin beta 16 (*Pcdhb16*) is a member of the protocadherin beta gene cluster. The protocadherin gene family encode proteins with an ectodomain comprising cadherin repeats. These protocadherin proteins have a single transmembrane domain and a distinct cytoplasmic domain, and can be further subdivided into clustered and non-clustered protocadherins. In the clustered protocadherins, there are alpha-, beta-, and gamma-protocadherin families while non-clustered protocadherins consists of individual protocadherins (1, 7, 9, 10, 11X, 11Y, 12, 17, 18, 19, and 20) (Gul et al., 2017). Studies have reported alpha-, and gamma- clustered protocadherins proteolysis by presenilins (Buchanan et al., 2010), whereas the non-clustered protocadherin-X, Y have been investigated as candidate genes for schizophrenia (Giouzeli et al., 2004; Kalmady and Venkatasubramanian, 2009). However, no study to date have been performed regarding the protocadherin beta 16 (*Pcdhb16*) as a schizophrenia-associated gene. The preexisting evidence of the role of protocadherin beta 16 pertains to associations with synaptic connectivity and neuronal networks, and is still under investigation (Junghans et al., 2008).

Arc has been mainly investigated for its roles in activity-driven synaptic plasticity and long-term memory consolidation (Rudinskiy et al., 2012). However, several literatures have examined the relevance between the *Arc* gene and schizophrenia (Manago and Papaleo, 2017). Particularly, schizophrenic mouse models showed reduced Arc expression (Takao et al., 2013; Takagi et al., 2015). In addition to alterations in gene expression, Arc knockout mice showed schizophrenic behaviors such as social impairment, impaired pre-pulse inhibition (which is a major characteristic observed in schizophrenia patients), and memory deficits (Manago et al., 2016). Furthermore, other possible roles of *Arc* has been suggested such as modulation of glutamatergic and dopaminergic systems which are key disrupted neurotransmitter systems in schizophrenia (Manago and Papaleo, 2017). Therefore, in the scope of the present study, altered Arc expression implicates that synaptic dysfunction via abnormal expression level of Arc may be involved in schizophrenic symptoms as well as memory loss and impaired cognitive function in AD.

Calponin-3 (*Cnn3*) is an actin-interacting protein and modulates postsynaptic currents and the size of dendritic spines (Rami et al., 2006). In a previous microarray study that was performed within the dorsolateral prefrontal cortex of schizophrenia patients, increased expression level of *Cnn3* was reported (Glatt et al., 2005). However, the roles of calponin-3 in schizophrenia have not been elucidated. Therefore, differentially expressed *Cnn3* in this study suggests the need for further investigation of the role of calponin-3 in schizophrenic symptoms of AD. This study suggests that the *Pcdhb16, Arc*, and *Cnn3* may be associated with synaptic functions on the basis of their GO terms. Together, considering the present study along with the aforementioned literature, abnormal expression levels of these genes in the cortex and hippocampus of AD point towards and provides evidence for synaptic dysfunction. This synaptic dysfunction in the cortex and hippocampus of AD may implicate schizophrenic symptoms of AD as well as memory loss and cognitive deficits.

As an important point to note, *Arc* and *Paxbp1* were expressed in opposite directions at 7 and 18 months. Expressions of Arc and Paxbp1 at 7 month were increased while these expressions at 18 month were decreased (**Figure 5**). This complex pattern of expression may have originated from the characteristics of these genes in the pathological conditions of AD. According to current literature, *Arc* expression in AD patients and mice showed discrepancies in results (Kerrigan and Randall, 2013). Furthermore, the expression level of *Arc* in the same AD mice changed depending on their ages (Kerrigan and Randall, 2013). One recent study with APP/PS1 AD mice provided a plausible explanation to these discrepancies (Rudinskiy et al., 2012). Amyloid plaques may elicit locally aberrant increase of Arc expression in active neurons. However, this study also observed a significant decrease of Arc expression in active neurons near amyloid plaques of mice. Due to unestablished role of Arc in AD, the Arc expression pattern in this study mice needs to be elucidated with definite experimental designs as a part of a future study.

PAX binding protein 1 (*PAXBP1*) is an adaptor protein linking the transcription factor PAX3 and PAX7 to the H3K4 histone methyltransferase complex. Although DNA hypo-methylation *in vitro* and higher levels of DNA methylation *in vivo* as epigenetic alterations in AD have been reported (Sanchez-Mut and Graff, 2015), roles of target genes of PAX3 and PAX7 such as Cdc20 and Id3 have not been researched in neither AD nor MDD (Diao et al., 2012). Therefore, we can restrictively interpret the opposite expression of PAXBP1 as potential roles in AD pathology based on epigenetic mechanisms with the need of further elucidation on the role of PAXBP1 in AD and MDD.

Until recently, NPS in AD have received less attention than memory loss and cognitive function deficits in AD. This big data analysis of genes associated to NPS in the AD animal model reveals the need for additional studies of NPS in AD. In the future, studies elucidating the underlying causal molecular mechanisms of NPS in both AD patients and AD animal models are needed.

## Materials and Methods

### Animals

We used PSEN dKO mice with partial deletion of PSEN1 in the forebrain and PSEN2 null. The wild type and PSEN dKO mice were fed *ad libitum*, and 2–4 animals were housed in a cage under the following cage conditions: constant humidity (45–55%), temperature (21–23°C), and 12 h light-dark cycle in a laboratory breeding room at the Korea Institute of Science and Technology (KIST). All procedures regarding the use and the handling of animals were approved by the Institutional Animal Care and Use Committee (IACUC) of KIST. All procedures were performed in accordance to the institutional guidelines and regulations on animal care and use from the IACUC.

### Preparation of Microarray Samples and the Number of Animals

The total number of animals used in the microarray were 14 mice. Specifically, the number of WT and PSEN dKO mice were eight and six respectively, and for each age group (mice of 7, 18 months), the number of tissue that were analyzed using microarray were eight and six, respectively. Thus, the number of WT and PSEN dKO mice in their respective age groups were: four WT mice of 7 and 18 months, four PSEN dKO mice of 7 months, and two mice for the PSEN dKO mice of 18 months. Excluding the group of PSEN dKO mice of 18 months, two samples were pooled for microarray, resulting in four groups with two samples for the microarray.

### RNA Preparation and Microarray

The wild type and PSEN dKO mice were sacrificed at the ages of 7 and 18 months. The complete mouse brain structure was removed and immediately prepared by being frozen on dry ice. The cortex and hippocampus were dissected and stored at -80°C until RNA isolation. Total RNA was isolated using the Trizol reagent. Messenger RNA expression profiling was performed using Affymetrix Mouse Genome 430 2.0 array chip containing 764,885 probe sets from 28,132 genes (Ensembl) or from 19,734 putative full-length transcripts (GenBank and Ref Seq). The microarray files can be accessed from Dryad Digital Repository^3^.

### KEGG Pathway

The 93, 11, and 69 DEGs within GO terms related to BIP, SCZ, and MDD were matched onto the functional annotation website, DAVID 6.8 (Database for Annotation, Visualization and Integrated Discovery). We uploaded the gene list using officially gene symbols at DAVID website, selected mus musculus as species, and then selected KEGG pathway with a cut off value of *p*-value < 0.05^4^ (Huang da et al., 2009a,b).

### Gene Ontology Annotation

We used 14 GO terms among 20 pathways (Network and Pathway Analysis Subgroup of Psychiatric Genomics Consortium, 2015) excluding cancer related pathways (Endometrial cancer, Prostate cancer, Thyroid cancer, and Glioma). Although we focused on neuropsychiatric disorders and AD, the KEGG analysis also revealed the relationship between cancer and AD in our study (**Supplementary Material S2**).

We downloaded GO annotations (GO version 2017-09-19) from the QuickGO GO ontology database^5^ (Binns et al., 2009). We used GO terms significantly relevant with the three disorders which were identified by previous research (Network and Pathway Analysis Subgroup of Psychiatric Genomics Consortium, 2015): BIP [GO:0051568 Histone H3-K4 methylation, GO:0007129 (Chromosomal) synapsis, GO:0090066 Regulation of anatomical structure size, GO:0070192 Chromosome organization involved in meiosis], SCZ (GO:0014069 Postsynaptic density, GO:0045211 Postsynaptic membrane, GO:0043197 Dendritic spine, GO:0051568 Histone H3-K4 methylation, GO:0033267 Axon part), and MDD (GO:0008601 Protein phosphatase type 2A regulator activity, GO:0034330 Cell junction organization, GO:0043297 Apical junction assembly, GO:0045216 Cell-cell junction organization, GO:0031056 Regulation of histone modification). These annotations were download under the QuickGO taxon criteria: Mus musculus (10090). Also, the QuickGO child term, “is a” condition was used. These annotations were also filtered by qualifiers: “part_of,” “involved_in,” “enables,” “contributes_to,” and “colocalizes_with.”

### Excel Based Differentially Expressed Gene Analysis (ExDEGA)

We used the ExDEGA software (ebiogen) in order to analyze separate genes within GO terms that were associated with BIP, SCZ, and MDD. ExDEGA is an analysis tool that facilitates the analysis of numerous data from microarray according to classified GO terms. Via the ExDEGA, we selected 2558 of a total of 45,037 genes. Within these 2558 genes, we defined significantly changed genes as genes with a *p*-value < 0.05 and a fold change of 1.3 compared to control mice. To correct for false positives we applied the Bonferroni and Benjamini-Hochberg correction methods as *post hoc* analyses. These correction identified only one gene (Cnn6) that was significantly altered, making further analysis impossible. Therefore, all analyses were conducted with the cut-off value (*P* < 0.05).

## Ethics Statement

We used PSEN dKO mice with partial deletion of PSEN1 in the forebrain and PSEN2 null. The wild type and PSEN dKO mice were fed *ad libitum*, and 2–4 animals were housed in a cage under the following cage conditions: constant humidity (45–55%), temperature (21–23°C), and 12 h light-dark cycle in a laboratory breeding room at the Korea Institute of Science and Technology (KIST). All procedures regarding the use and the handling of animals were approved by the Institutional Animal Care and Use Committee (IACUC) of KIST. All procedures were performed in accordance to the institutional guidelines and regulations on animal care and use from the IACUC.

## Author Contributions

H-II and Y-PT designed the study. H-II carried out the RNA isolation and microarray, and reviewed and commented on the manuscript. SH and TK analyzed, interpreted data, and drafted the manuscript. HH and YK discussed and edited the manuscript. All authors read and approved the final manuscript.

## Conflict of Interest Statement

The authors declare that the research was conducted in the absence of any commercial or financial relationships that could be construed as a potential conflict of interest.

## Footnotes

1 http://www.alz.org/care/alzheimers-dementia-depression.asp#symptoms

2 http://www.alz.org/care/alzheimers-dementia-hallucinations.asp

3 https://doi.org/10.5061/dryad.408d6f0

4 http://www.genome.ad.jp/kegg

5 www.ebi.ac.uk/QuickGO/

## Supplementary Material

SUPPLEMENTARY MATERIAL S1 Gene lists of DEGs within GO terms of BIP, SCZ, and MDD.

SUPPLEMENTARY MATERIAL S2 KEGG pathways with DEGs within GO terms related to BIP, SCZ, and MDD.
